# Sintilimab for relapsed/refractory extranodal NK/T cell lymphoma: a multicenter, single-arm, phase 2 trial (ORIENT-4)

**DOI:** 10.1038/s41392-021-00768-0

**Published:** 2021-10-27

**Authors:** Rong Tao, Lei Fan, Yongping Song, Yu Hu, Wei Zhang, Yafei Wang, Wei Xu, Jianyong Li

**Affiliations:** 1 Department of Hematology, Xinhua Hospital, Shanghai Jiao Tong University School of Medicine, Shanghai, China; 2 The First Affiliated Hospital of Nanjing Medical University, Jiangsu Province Hospital, Collaborative Innovation Center for Cancer Personalized Medicine, Nanjing, China; 3 The Affiliated Cancer Hospital of Zhengzhou University and Henan Cancer Hospital, Zhengzhou, China; 4 Union Hospital Tongji Medical College Huazhong University of Science and Technology, Wuhan, China; 5 Peking Union Medical College Hospital, Beijing, China; 6 Tianjin Medical University Cancer Institute and Hospital, Tianjin, China

**Keywords:** Haematological cancer, Haematological cancer

## Abstract

This study (ORIENT-4) aimed to assess the efficacy and safety of sintilimab, a humanized anti-PD-1 antibody, in patients with relapsed/refractory extranodal NK/T cell lymphoma (r/r ENKTL). ORIENT-4 is a multicenter, single-arm, phase 2 clinical trial (NCT03228836). Patients with r/r ENKTL who failed to at least one asparaginase-based regimen were enrolled to receive sintilimab 200 mg intravenously every 3 weeks for up to 24 months. The primary endpoint was the objective response rate (ORR) based on Lugano 2014 criteria. Twenty-eight patients with r/r ENKTL were enrolled from August 31, 2017 to February 7, 2018. Twenty-one patients (75.0%, 95% CI: 55.1–89.3%) achieved an objective response. With a median follow-up of 30.4 months, the median overall survival (OS) was not reached. The 24-month OS rate was 78.6% (95% CI, 58.4–89.8%). Most treatment-related adverse events (TRAEs) were grade 1–2 (71.4%), and the most common TRAE was decreased lymphocyte count (42.9%). Serious adverse events (SAEs) occurred in 7 (25.0%) patients, and no patient died of adverse events. Sintilimab is effective and well tolerated in patients with r/r ENKTL and could be a novel therapeutic approach for the control of ENKTL in patients.

## Introduction

Extranodal natural killer (NK)/T cell lymphoma (ENKTL) is a highly aggressive subtype of non-Hodgkin lymphoma (NHL) with a high incidence in Asia and Latin America.^1^ In China, ENKTL accounts for 6.6% of all NHL and 28.1% of peripheral T lymphomas (PTCLs).^2^ The 5-year overall survival (OS) of patients with ENKTL is 40–50%.^1^

ENKTL shows a poor response to conventional anthracycline-based chemotherapy due to the high expression of the multidrug resistance P-glycoprotein. Asparaginase-based regimens are effective for ENKTL with an objective response rate (ORR) of 67–79% and a complete response (CR) rate of 45–61%.^3–5^ Patients who failed asparaginase-based therapy have limited treatment options with a median survival of <6 months.^6^

Epstein–Barr virus (EBV) infection is an important etiological and prognostic factor for ENKTL.^7^ Overexpression of PD-L1 induced by EBV infection is a potential mechanism for ENKTL to avert immune surveillance, and anti-PD-1 antibodies in patients with relapsed/refractory (r/r) ENKTL have shown potential efficacy.^8^ Anti-PD-1 monoclonal antibody (mAb) can rescue T cell viability inhibited by EBV-positive diffuse large B cell lymphoma.^9,10^ Two retrospective studies with limited sample size showed that pembrolizumab was active in r/r ENKTL with ORRs of 100% (*n* = 7)^11^ and 57.1% (*n* = 7),^12^ respectively. Two case reports also demonstrated the efficacy of anti-PD-1 mAb in r/r ENKTL after asparaginase-based therapy.^13,14^ Recently, there have been a study reporting the efficacy and safety of PD-L1 antibody—avelumab monotherapy for r/r NKTCL—that showed an ORR of 38% in 21 patients, with an CR rate of 24%.^15^ However, there is scarce clinical evidence for the antitumor activity of PD-1 inhibitors in patients with ENKTL.

Sintilimab is a recombinant humanized anti-PD-1 mAb that binds to human PD-1. Compared with pembrolizumab or nivolumab, sintilimab has a different binding site and potentially greater affinity against PD-1 according to preclinical data.^16^ Sintilimab has demonstrated clinical benefit in various cancers.^17,18^

The present prospective phase 2 study was conducted to evaluate the efficacy and safety of sintilimab against r/r ENKTL.

## Results

### Baseline characteristics

From August 31, 2017, to February 7, 2018, 34 patients were screened and 28 patients were eventually enrolled. Patients were excluded because the lesion was <15 mm or not visible by ^18^F-fluorodeoxyglucose positron emission tomography (^18^FDG-PET) (*n* = 3), insufficient organ or bone marrow function (*n* = 1), uncontrolled concurrent disease (*n* = 1), and active hepatitis B (*n* = 1) (Supplementary Fig. S1).

The median age of the patients was 37 years (range: 19–65), and 17 (60.7%) were male. Nineteen patients (67.9%) were stage IV, 25 (89.3%) patients had an Eastern Cooperative Oncology Group (ECOG) performance status (PS) of 1 and 2. Patients received a median of 3 (Q1–Q3, 2.0–4.5) prior lines of chemotherapy, and all were relapsed or refractory to previous asparaginase-based treatment. Twenty-two (78.6%) patients previously received radiotherapy, and 2 (7.1%) underwent autologous stem cell transplantation (Table 1).^19^

**Table 1:** Baseline characteristics of the patients

Characteristics	Total (*n* = 28)
Age, years	
Mean ± standard deviation	39.8 ± 12.67
Median (range)	37 (19–65)
Sex, *n* (%)	
Male	17 (60.7)
Female	11 (39.3)
Ethnic group, *n* (%)	
Han	28 (100.0)
Time from first diagnosis, months	
Median (Q1–Q3)	22.0 (10.0–40.2)
ECOG PS, *n* (%)	
0	3 (10.7)
1	24 (85.7)
2	1 (3.6)
Previous lines of chemotherapy	
Median (Q1–Q3)	3.0 (2.0–4.5)
≥3, *n* (%)	15 (53.6)
Previous radiotherapy, *n* (%)	
Yes	22 (78.6)
No	6 (21.4)
Previous autologous stem cell transplantation, *n* (%)	
None	26 (92.9)
Once	2 (7.1)
Asparaginase-based treatment outcome	
Refractory	12 (42.9)
Relapsed	16 (57.1)
Bone marrow involvement, *n* (%)	
No	22 (78.6)
Yes	6 (21.4)
B symptoms, *n* (%)	
Presence	24 (85.7)
Absence	4 (14.3)
Lactate dehydrogenase, *n* (%)	
Normal	10 (35.7)
Increased	18 (64.3)
Plasma EBV, *n* (%)	
Positive	8 (28.6)
Negative	20 (71.4)
Ann Arbor stage, *n* (%)	
I	2 (7.1)
II	7 (25.0)
III	0
IV	19 (67.9)
PINK, *n* (%)	
Low	9 (32.1)
Intermediate	11 (39.3)
High	8 (28.6)
PINK-E, *n* (%)	
Low	16 (57.1)
Intermediate	9 (32.1)
High	3 (10.7)

*ADA* anti-drug antibody, *EBV* Epstein–Barr virus, *ECOG PS* Eastern Cooperative Oncology Group performance status, *PD* progressive disease, *PINK* Prognostic Index for Natural Killer Lymphoma, risk factors include: age >60 years, stage III or IV, distant lymph node involvement, and non-nasal type disease;^22^ *PINK-E* Prognostic Index for Natural Killer Lymphoma with EBV DNA, risk factors include: age >60 years, stage III or IV, distant lymph node involvement, non-nasal type disease, and EBV DNA^22^

### Response

By February 28, 2020, the median follow-up was 30.4 months (range: 27.5–31.9). Median duration of treatment was 24.2 months (range: 1.4–33.3) and a median number of received doses was 35 (range: 2–48). Five patients who were initially determined as progressive disease (PD) finally showed a response (they were considered as pseudo-progression), and the ORR (*n* = 21) was in fact 75.0% (95% confidence interval (CI): 55.1–89.3) when including the 5 patients who experienced pseudo-progression prior to response. The CR and partial response (PR) rates were 21.4 and 53.6%, respectively. The disease control rate (DCR) was 85.7% (95% CI: 67.3–96.0%), including 5 patients who experienced pseudo-progression prior to response (Table 2). The first response evaluation (at 6 weeks) after enrollment of 26 patients showed 14 responders (CR *n* = 1, and PR *n* = 13), 4 patients having stable disease (SD), and several patients with early PD (*n* = 8). Enrollment was paused due to these PD cases, but treatment was continued. Table 2 presents the responses during the whole trial. Since the lower limit of 95% CI of ORR was above the pre-specified 30% threshold, the H1 hypothesis for the efficacy of sintilimab was verified, and the enrollment for the present trial was terminated.

**Table 2:** Efficacy evaluation of sintilimab during the whole trial^a^

Efficacy outcomes	Primary endpoint	Cycle 3	Cycle 6	Cycle 9	Cycle 13	Cycle 17
	Last follow-up	6 weeks	15 weeks	24 weeks	36 weeks	48 weeks
	*n* = 28	*n* = 26	*n* = 25	*n* = 20	*n* = 20	*n* = 20
CR	6 (21.4%)	1 (3.8%)	1 (4.0%)	2 (10.0%)	3 (15.0%)	3 (15.0%)
PR	15 (53.6%)	13 (50.0%)	7 (28.0%)	4 (20.0%)	0 (0.0%)	0 (0.0%)
SD	3 (10.7%)	4 (15.4%)	8 (32.0%)	6 (30.0%)	17 (85.0%)	17 (85.0%)
PD	3 (10.7%)	8 (30.8%)	9 (36.0%)	7 (35.0%)	0 (0.0%)	0 (0.0%)
Unevaluable	1 (3.6%)	0 (0.0%)	0 (0.0%)	1 (5.0%)	0 (0.0%)	0 (0.0%)
Objective response (CR + PR)	21 (75%)	53.8%	32.0%	30.0%	15.0%	15.0%
Objective response rate (95% CI)	55.1–89.3%	33.4–73.4%	14.9–53.5%	11.9–54.3%	3.2–37.9%	3.2–37.9%
Disease control (CR + PR + SD)	24 (85.7%)	69.2%	64.0%	60.0%	100.0%	100.0%
Disease control rate (95% CI)	67.3–96%	48.2–85.7%	42.5–82.0%	36.1–80.9%	83.2–100.0%	83.2–100.0%

*CR* complete response, *DCR* disease control rate, *ORR* objective response rate, *PD* progressive disease, *PR* partial response, *SD* stable disease

^a^Binomial distribution was adopted for ORR and disease control rate to calculate the confidence intervals (CIs)

**Fig. 1:**
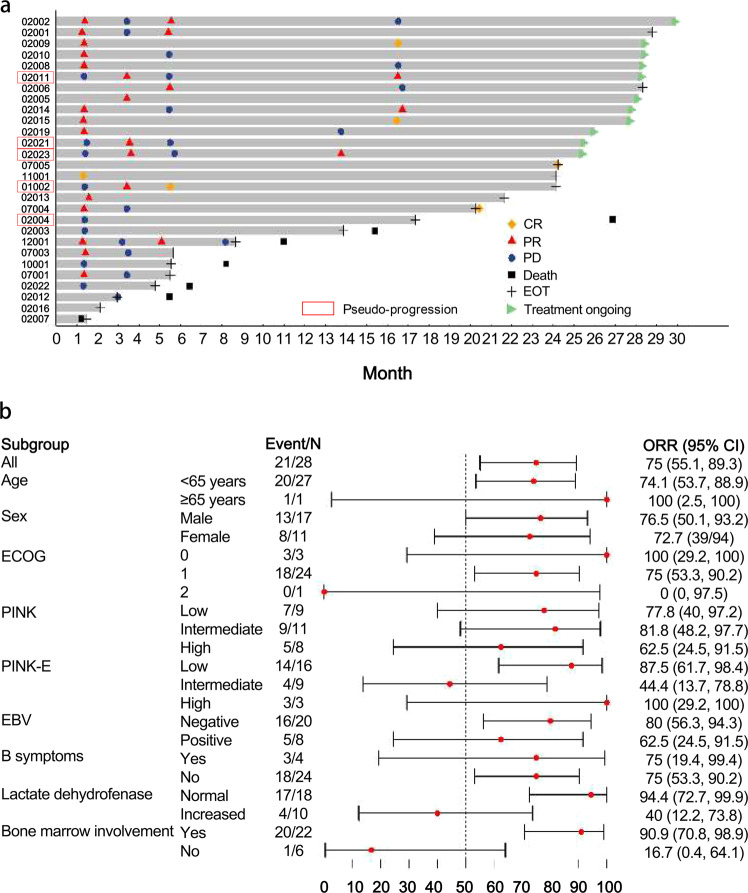
Response to sintilimab in patients with relapsed/refractory NK/T cell lymphoma. **a** Analysis of time to response and duration of response. Patient 02004 was evaluated as stable disease after experiencing pseudo-progression. **b** Subgroup analysis of ORR

The median time to response (TTR) was 1.3 months (95% CI: 1.3–3.4), and the median duration of response (DOR) was 4.1 months (95% CI: 2.1–15.2) (Fig. 1a). Subgroup analyses suggested that normal lactate dehydrogenase (LDH, *n* = 18, ORR = 94.4%, 95% CI: 65.3–98.6%) and absence of bone marrow involvement (*n* = 22, ORR = 90.9%, 95% CI: 59.7–94.8%) were associated with more favorable ORR (Fig. 1b).

### Survival

**Fig. 2:**
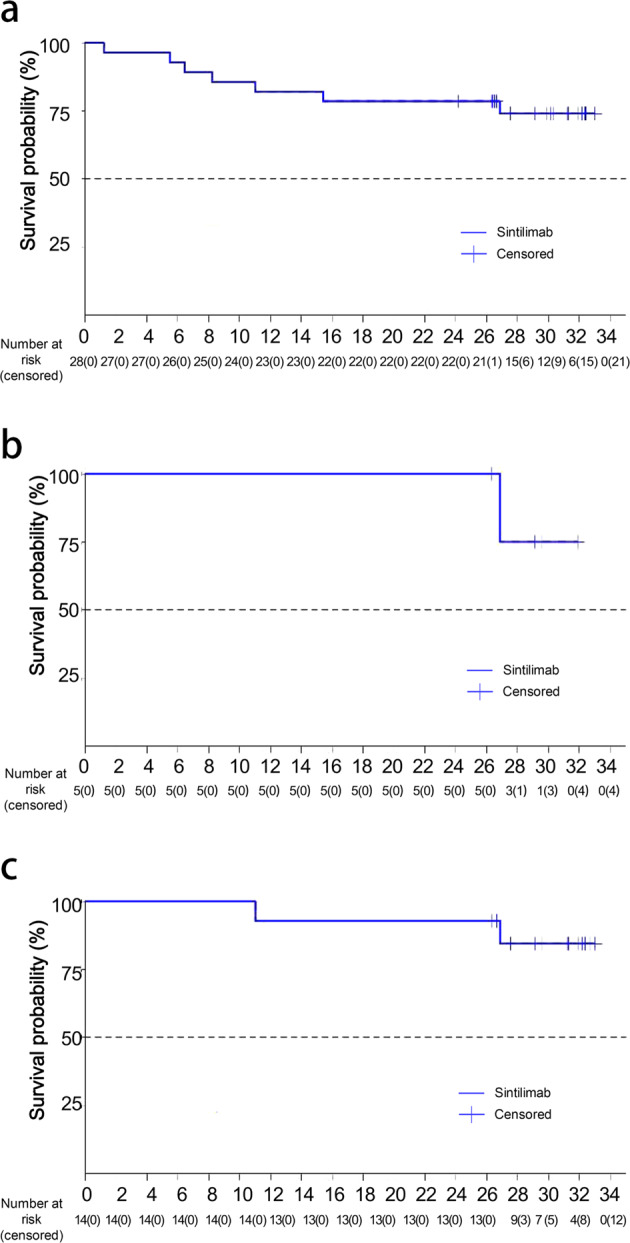
Overall survival with sintilimab in patients with NK/T cell lymphoma. **a** Overall survival in all patients (28 patients) (*X*-axis refers to time of follow-up in months). **b** Overall survival in patients with pseudo-progression (5 patients) (*X*-axis refers to time of follow-up in months). **c** Overall survival in patients with the response pattern displayed as transient flares in different nodal groups without overall progression in the original target lesions (14 patients) (*X*-axis refers to time of follow-up in months)

**Fig. 3:**
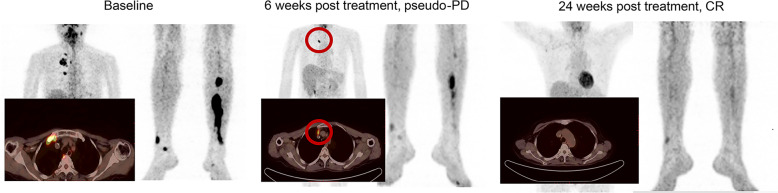
Patterns of radiologic tumor pseudo-progression after sintilimab. This picture shows CT imaging of a 59-year-old female patient in stage IV involving the head and neck area, mediastinum, and shank (left). This patient was first diagnosed with ENKTL in 2017 and previously received three cycles of L-asparaginase plus gemcitabine and oxaliplatin (GELOX) followed by local radiotherapy (50 Gy). On January 31, 2018, after progressive disease, the patient participated in ORIENT-4 and received sintilimab 200 mg Q3W. Six weeks after treatment, most lesions regressed, while a new lesion appeared on the right mediastinal pleura (middle), which had not been observed before. Twenty-four weeks after treatment, CT imaging showed further regression of right mediastinal pleura lesions, with the appearance of new lesions in week 6; the patients achieve a complete response (right). The areas marked by red cycles were new lesions

Till data cut-off, seven patients had death events. The 1-year OS rate was 82.1% (95% CI: 62.3–92.1%) and 2-year OS rate was 78.6% (95% CI: 58.4–89.8%) (Fig. 2a). Five (17.9%) patients had pseudo-progression. In these patients, the 1- and 2-year OS rates were both 100.0% (95% CI: 100.0–100.0), and the median OS was not reached (Fig. 2b). Fourteen (50.0%) patients, including 5 who had pseudo-progression, showed a pattern that despite new lesions or increased ^18^FDG-PET uptake might be observed in asymptomatic patients after sintilimab administration, decreased uptake could be observed subsequently (Fig. 3). Among these patients, the OS rates at 1- and 2-year were both 92.9% (95% CI: 59.1–99.0; Fig. 2c).

### Historical controls

In the retrospective analysis, 46 patients were identified with r/r ENKTL failing to asparaginase-based regimens. In these patients, ORR was 32.8%, and the median OS was 4.8 months (Supplementary Fig. S2).

### Safety

Twenty-seven (96.4%) patients experienced at least one treatment-related adverse event (TRAE), with the most common TRAEs being decreased lymphocyte count (46.5%), pyrexia (42.9%), and decreased white blood cell count (35.7%). Most TRAEs were grade 1–2, and only one patient experienced grade 4 TRAE (diabetes). No grade 5 TRAE was reported (Table 3). Seven (25.0%) patients reported serious AEs (SAEs), including grade 3 pulmonary infection, anaphylactic shock, acute pancreatitis, gastrointestinal hemorrhage, intervertebral disc disorder, localized infection, ketoacidosis, and grade 2 pyrexia. Only ketoacidosis was related to sintilimab by the investigator. Two patients reported TRAE (diabetes and platelet count decreased) led to treatment discontinuation. No positive anti-drug antibody (ADA) or infusion-related AE was reported.

**Table 3:** Most common TRAEs

Treatment-related adverse events, *n* (%)	All patients (*N* = 28)	
	Grade 1–2	Grade 3
Any TRAE	17 (60.7)	11 (39.3)
Lymphocyte count decreased	12 (42.9)	2 (7.1)
White blood cell count decreased	12 (42.9)	0
Pyrexia	12 (42.9)	0
Hypothyroidism	9 (32.1)	0
Blood thyroid-stimulating hormone increased	7 (25.0)	0
Blood glucose increased	7 (25.0)	0
Upper respiratory tract infection	6 (21.4)	1 (3.6)
Hemoglobin decreased	6 (21.4)	0
Platelet count decreased	5 (17.9)	1 (3.6)
Globulin increased	5 (17.9)	0
Blood alkaline phosphatase increased	5 (17.9)	0
Nasosinusitis	5 (17.9)	0
Urinary tract infection	5 (17.9)	0

Listed are any TRAEs occurring in ≥15% patients

*TRAE* treatment-related adverse event

### Quality of life

Quality of life of the enrolled patients was significantly improved, starting at week 15. For the EuroQol-5-Dimensional-5-level (EQ-5D-5L) index, the mean score increased from 0.8 at baseline to 0.9 at week 15 (*P* < 0.05) and then remained at 0.9 to Week 96 (Supplementary Fig. S3A). For EQ-5D-5L Vas, the mean score increased from 79.3 at baseline to 90.6 at week 24 (*P* < 0.0001) and then remained between 87.9 and 91.8 from weeks 24 to 96 (Supplementary Fig. S3B). For QLQ-C30, the mean score increased from 70.5 at baseline to 85.4 at week 24 (*P* < 0.0001) and then remained between 82.0 and 87.3 from weeks 24 to 96 (Supplementary Fig. S3C).

## Discussion

This phase 2 study showed that sintilimab was effective and well tolerated in patients with r/r ENKTL.

Originally, we planned to enroll 20–60 patients and a minimum sample size of 43 was required based on the statistical analysis. However, multiple early PD cases were observed after enrolling 28 patients, and the evaluation on response became difficult per Lugano 2014 criteria.

Therefore, enrollment was paused and treatment was continued to confirm the true nature of the pseudo-progression according to the investigator’s discretion. After 3 months of observation and in-depth analysis of 28 patients, sintilimab demonstrated a 75.0% ORR when taking pseudo-progression into account. Under the hypothesis that the 15 yet-to-be-enrolled patients would be non-responders, there would be 19 responders out of 43 overall patients (28 actually enrolled + 15 fictive), providing a 95% CI of 30.4–58.9% by Wilson’s method, where the lower boundary was still greater than the predefined 30% threshold. From the view of proof of concept, the efficacy of sintilimab in r/r ENKTL patients was proved.

The standard treatment for ENKTL after the failure of the asparaginase-based regimen has not been established.^20^ The multidrug resistance-independent DeVIC regimen has been suggested for ENKTL, but this strategy is currently used to combine with radiotherapy, and there is no data regarding r/r ENKTL.^21^ Gemcitabine-containing regimen for r/r ENKTL failing to L-asparaginase-based therapy achieved an ORR of 40% but had a dismal long-term outcome.^22^ No data are available about the application of other drugs such as histone deacetylase inhibitors and anti-CD30 mAbs for ENKTL, which are effective in PTCL.

In the ORIENT-4 study, the ORR for sintilimab in r/r ENKTL was 75.0% (95% CI: 55.1–89.3%), and the median DOR was 4.1 months (range: 2.1–15.2). In previous small-scale studies (*n* = 7), the ORRs of anti-PD-1 antibody in r/r ENKTL ranged from 57.1 to 100%.^12,13^ The retrospective analysis based on the clinical data in 46 patients with r/r ENKTL here showed a lower ORR of 32.8%. A recently published phase 2 study conducted by Professor Won Seog Kim’s team showed that anti-PD-L1 mAb—avelumab monotherapy—achieved an ORR of 38% and CR rate of 24% in r/r ENKTL patients. Comparing this study to the present study, the sample was similar (21 vs 28), the CR rate was comparable (24 vs 21.4%), but the ORR was significantly different (38 vs 75%); one possible reason might be that sintilimab was administered after PD at the investigator’s discretion, and most patients who experienced PD eventually received sintilimab. This was significantly different from the phase 2 study of avelumab, in which patients withdrew from study immediately after PD.^15^ No previous study reported DOR and long-term survival of anti-PD-1/PD-L1 antibody in r/r ENKTL. Efficacy data of ORIENT-4 demonstrated that sintilimab was effective in patients with r/r ENKTL.

In the present study, the long-term efficacy of sintilimab was favorable in r/r ENKTL, with a 1-year OS rate of 82.1% and a 2-year OS rate of 78.6%, and the median OS was not reached. Previous retrospective analysis showed that the median OS was 6.4 months in comparable patient cohort of r/r ENKTL^6^ and r/r PTCL (including ENKTL, 6.5 months).^23^ Therefore, these results suggested that the current salvage treatments for r/r ENKTL are inadequate, and sintilimab could be one helpful treatment strategy. We suggest that the follow-up time could be 16–34 weeks for the evaluation of ORR, which lasts for about 6 months. Still, for r/r ENKTL patients with no evidence of PD, a longer follow-up time could have a higher clinical significance.

During this study, 5 (17.9%) patients were observed having pseudo-progression, and 14 (50.0%) patients showed a fluctuant response. Of all the patients with pseudo-progression (100%), their 1- and 2-year OS rates were relatively greater than the overall population. In addition, the median OS was not reached, demonstrating no association between pseudo-progression or fluctuant response and an inferior OS. This suggests that the phenomenon of pseudo-progressions or fluctuant response should be taken into account during sintilimab treatment and must be confirmed before treatment alteration. Failing to confirm the true nature of the apparent PD could lead to early cessation of effective treatment and decrease possible benefits of patients.

Pseudo-progression is not rare when treating patients with immune checkpoint inhibitors. In a previous study on solid tumors treated with pembrolizumab (*n* = 655), the early pseudo-progression and delayed pseudo-progression was reported in 5 and 3% of patients, respectively.^24^ Kwong et al. reported one of the seven pseudo-progression cases in patients with r/r ENKTL treated with pembrolizumab.^13^ In the present study, the response pattern was displayed as transient flares in different nodal groups without overall progression, which was consistent with the response pattern observed with nivolumab for Hodgkin lymphoma.^25^ For the study design in 2016, the Lugano 2014 criteria were recommended for NHL evaluation, but it lacked the criteria for pseudo-progression in lymphoma response assessment. In this study, it was observed that pseudo-progression or fluctuant response is not rare in ENKTL, thus Lugano 2014 criteria may not be the best option for this trial. Although 16–24 weeks is the recommended time point for ORR evaluation after standard treatment in patients with NHL, long-term observation and an extended time point of 6 months and 1 year should be recommended for ORR evaluation in patients with r/r ENKTL lacking obvious disease progression. In this study, several patients achieved PR or CR after >1 year of treatment. Nevertheless, differentiating pseudo-progression from true progression is a major challenge since there is no uniform definition of pseudo-progression.^11,26–28^ Additional studies are necessary to identify reliable markers, and our limited experience of identification of pseudo-progression are: (1) repeated imaging examination at longer evaluation time point; (2) serological markers like LDH, ferritin, and sCD25; (3) circular EBV DNA copy number load; (4) clinical symptoms and quality of life scores. Regarding the time point for the evaluation of treatment efficacy, NHL has suggested that imaging evaluation should be performed at 4–6 months. Nevertheless, the findings of this study suggest that the treatment with checkpoint inhibitors in ENKTL might induce a relatively long-time pseudo-progression. The definite time point for the evaluation of treatment efficacy could not be concluded in this study. Our experience suggests that the time point of evaluation should be later than 6 months.

In the present study on sintilimab monotherapy, the DCR (85.7%) was excellent, but the deep response rate (CR 21.4%) was relatively low, suggesting that combination therapy might be needed to improve the CR rate. Several studies are ongoing on the use of anti-PD-1 antibodies in combination with chemotherapy (NCT04004572, NCT03701022, and NCT03936452), and we look forward to their results for further information.

In the present study, all patients experienced at least one AE, but no grade 5 AE was observed. The most common TRAEs were decreased lymphocyte count (46.4%), pyrexia (42.9%), and decreased white blood cell count (35.7%). This was globally consistent with the results of the ORIENT-1 study,^17^ in which 93% of the patients had at least one AE, and 15% had SAEs.^17^ In addition, this safety profile was generally more favorable than that observed for chemotherapy in patients with ENKTL, in which grade 3–4 AEs were more common than those observed with sintilimab.^3–5^ Overall, the safety profile was consistent with that of other PD-1 targeted therapies in similar patients.^11,17,18,29,30^

The present study also has some limitations. First, it was a single-arm study without any direct control. Only historical controls from a single center were used. Second, the outcomes were assessed by investigators, which might have a bias on the evaluation. Third, the response criteria were based on Lugano 2014, which might not be the best option for patients with ENKTL treated with an immune checkpoint inhibitor. Fourth, the patients received a wide variety of treatments after this study. The treatments were too diverse for further analysis.

A major limitation is that the study did not reach its pre-specified sample size. Indeed, the study was stopped temporarily due to the large number of cases with PD at 6 weeks (*n* = 9), of whom 4 cases were finally ruled out to be pseudo-progression, and the censoring time was already reached when the patients were confirmed with pseudo-progression. Therefore, only 28 patients were enrolled in this study. In addition, response evaluation at 6 weeks was probably not adequate for determining the ORR. Still, according to the actual ORR in this study, as well as *α* = 0.05, power = 80%, and historical control of 30%, a post hoc power analysis showed that the sample size required would be 10. If the historical control were 40%, the sample size required would be 23. In this study, 28 patients were enrolled when the enrollment was stopped, which could meet the statistical requirement. As this study is sufficient to reach a conclusion, no further studies were performed.

In conclusion, sintilimab is effective and well tolerated in patients with r/r ENKTL and can be a promising treatment option. Further studies are necessary to determine the exact benefit for these patients treated with sintilimab-based regimens.

## Material and methods

### Study design and participants

This multicenter single-arm phase 2 clinical study (ORIENT-4) was conducted at six hospitals in China (NCT03228836). Patients aged between 18 and 70 years, with histopathologically confirmed ENKTL and with at least one measurable lesion (>15 mm or positive ^18^FDG-PET uptake) were enrolled. Other major inclusion criteria were ECOG PS of 0–2, adequate organ and bone marrow functions, had received at least one prior asparaginase-based chemotherapy (stage I/II patients must have been treated with local radiotherapy), and either relapsed or refractory to previous treatments. Relapse is defined as new lesions at the primary location or other sites after achieving CR; refractory is defined as any of the following: PD after two treatment cycles, not achieving a PR, or CR after four or six treatment cycles, respectively.

The major exclusion criteria included: aggressive NK cell leukemia, primary or secondary central nervous system lymphoma, severe hemophagocytic syndrome at initial diagnosis of ENKTL-NT, and previous exposure to any checkpoint inhibitors. The complete eligibility criteria are shown in Supplementary Materials.

Retrospective data of patients with r/r ENKTL to asparaginase-based regimens were collected from the database of Jiangsu Province Hospital (Nanjing, China) as historical controls. The inclusion criteria were (1) patients with r/r ENKTL; (2) failed L-asparaginase therapy.

This study was approved by the ethics committee of each participating center and was registered at www.clinicaltrials.gov (NCT03228836). It was conducted in accordance with the principles of the Declaration of Helsinki and the Good Clinical Practice guideline. All patients provided written informed consent prior to any study procedure.

### Procedures

The eligible patients received sintilimab 200 mg intravenously every 3 weeks until PD, death, intolerable toxicity, or withdrawal of informed consent, up to 24 months. Treatment beyond PD was allowed according to the investigator’s discretion.

The patients were evaluated for efficacy using ^18^FDG-PET at baseline and weeks 6, 15, and 24. Contrast-enhanced computed tomography was used at baseline and weeks 24, 36, and 48 and every 24 weeks thereafter. Efficacy was assessed according to the Lugano 2014 standard.^31^ The best overall response was the best response recorded from the start of the study until progression/relapse. Safety was monitored throughout the study until 90 days after the last dose of sintilimab. The quality of life was analyzed at the initial dose, at weeks 6, 15, 24, 36, and 48, and every 24 weeks thereafter. The blood samples for ADA and neutralizing antibody (NAb) were collected at baseline and just before sintilimab administration at weeks 3 and 9 and every 12 weeks thereafter. NAb has been reported for sintilimab.^16^

During the trial, for patients with PD, the investigators comprehensively judged from their radiographic results, laboratory results, and clinical performance, and some were evaluated as being in pseudo-progression. For these patients, treatment was given continuously, and investigators continue to observe and follow up these patients in accordance with the requirements of the clinical trial design.

### Outcomes

The primary endpoint was the ORR (CR + PR) of sintilimab monotherapy for r/r ENKTL. The secondary endpoints included CR and PR rates, DCR (CR + PR + SD), TTR, DOR, 1-year OS, safety, and quality of life. Reported adverse events were graded based on the Common Terminology Criteria for Adverse Events (Version 4.03). The quality of life was evaluated using the EQ-5D-5L scale and the European Organization for Research and Treatment of Cancer Quality-of-Life Questionnaire-Core 30 (EORTC QLQ-C30).

### Statistical analysis

It was planned to enroll 20–60 r/r ENKTL patients. The ORR was set to ≤30% in the null hypothesis using a two-sided *α* of 0.05. To reject the null hypothesis and achieve an expected ORR of 50% with an 80% power, a minimum of 43 patients was required. If the lower boundary of the ORR 95% CI was >30%, the efficacy of sintilimab monotherapy for r/r ENKTL was demonstrated. The choice of 30% ORR was based on the historical ORR data for r/r NK/T and r/r PTCL. Patients with r/r ENKTL reported a 40% ORR after gemcitabine-based treatment^22^ and achieved a 15% ORR after chidamide monotherapy.^32^ Patients with r/r PTCL obtained an ORR of 28% after asparaginase-based regimen in a Chinese population.^33^

The binomial distribution was used to estimate ORR, CR, PR, and DCR and the 95% CIs. Kaplan–Meier method was applied to estimate the time-to-event endpoints (DOR and OS) and their 95% CI. For all analyses, *P* values <0.05 were considered statistically significant. All analyses were carried out using SAS 9.4 (SAS Institute, NY, USA).

## Supplementary information

Supplementary file

## Data Availability

All data or resources used in the paper are available by reasonable requirements to the corresponding authors L.F. (fanlei3014@126.com) and J.L. (jianyong.lijsh@outlook.com).
