# Supplementary material for: Sintilimab for relapsed/refractory extranodal NK/T cell lymphoma: a multicenter, single-arm, phase 2 trial (ORIENT-4)
Source: Signal Transduct Target Ther. 2021 Oct 27;6:365. doi: 10.1038/s41392-021-00768-0 (PMC8548511; doi:10.1038/s41392-021-00768-0)
Supplement: Supplementary file 1 — Supplementary file [file 41392_2021_768_MOESM1_ESM.docx]

**Supplementary Materials for**

**Sintilimab for relapsed/refractory** **extranodal NK/T-cell lymphoma:** **A multicenter, single-arm, phase 2 trial (ORIENT-4)**

Rong Tao ^1^, Lei Fan ^2*^, Yongping Song ^3^, Yu Hu ^4^, Wei Zhang ^5^, Yafei Wang ^6^, Wei Xu ^2^, Jianyong Li ^2*^

^1^ Department of Hematology, Xinhua Hospital, Shanghai Jiao Tong University School of Medicine, Shanghai, China

^2^ The First Affiliated Hospital of Nanjing Medical University, Jiangsu Province Hospital, Collaborative Innovation Center for Cancer Personalized Medicine, Nanjing, China.

^3^ The Affiliated Cancer Hospital of Zhengzhou University and Henan Cancer Hospital, Zhengzhou, China

^4^ Union Hospital Tongji Medical College Huazhong University of Science and Technology, Wuhan, China

^5^ Peking Union Medical College Hospital, Beijing, China

^6^ Tianjin Medical University Cancer Institute and Hospital, Tianjin, China

Correspondence to: fanlei3014@126.com (Lei Fan)

jianyong.lijsh@outlook.com (Jianyong Li)

**This PDF file includes:**

Supplementary Text

Figure S1 to S3

**Supplementary Text**

Inclusion and exclusion criteria

Inclusion criteria

1. Histologically confirmed ENKL-NT.
2. Relapsed or refractory ENKTL-NT. Being relapsed is defined as the presence of new lesions at the primary location or other sites after achieving CR; being refractory is defined as anyone of the following: PD after two treatment cycles, PR not achieved after four treatment cycles, or CR not achieved after six treatment cycles. Patients who do not respond or patients with relapsed disease or PD after autologous stem cell transplantation can enroll.
3. Must have been treated with asparaginase-based regimen (radiotherapy must be performed for stage I/II disease).
4. Long axis of a lesion >15 mm or ^18^FDG-PET uptake by the lesion.
5. Eastern Cooperative Oncology Group Performance Status (ECOG PS) scores of 0–2.
6. Signed the ICFs and are able to comply with the scheduled follow-up visits and related procedures required in the protocol.
7. Between the ages of >18 to <70 years.
8. Life expectancy ≥12 weeks.
9. Patients (female patients at childbearing age or male patients whose partners are at childbearing age) must take effective contraceptive measures during the entire course of the trial and within 90 days since the last dose of treatment (see Section 4.3).
10. Adequate organ and bone marrow functions, as defined below:

- Count of whole blood cells: absolute neutrophil count (ANC) ≥1·0 × 10^9^/L, platelet (PLT) count ≥50 × 10^9^/L, hemoglobin (HGB) ≥8·0 g/L; granulocyte colony-stimulating factor, PLT, or red blood cell transfusion has not been performed within 7 days prior to the test.
- Hepatic function: total bilirubin (TBIL) ≤1·5 × upper limit of normal (ULN), alanine aminotransferase (ALT) and aspartate aminotransferase (AST) ≤2·5 × ULN.
- Renal function: serum creatinine (Cr) ≤1·5 × ULN.
- Thyroid function: normal thyroid-stimulating hormone (TSH) at baseline, or abnormal TSH at baseline with normal T3/T4 and no symptoms.

Exclusion criteria

1. Patients with aggressive NK cell leukemia.
2. Patients with primary or secondary central nervous system lymphoma.
3. Patients with the severe hemophagocytic syndrome at initial diagnosis of ENKTL-NT.
4. Patients with pulmonary great vessel invasion.
5. Previous exposure to any anti-PD-1, anti-PD-L1, or anti-CTLA-4 antibodies.
6. Enrolled in another interventional clinical study, unless only involved in an observational study (noninterventional) or in the follow-up phase of an interventional study.
7. Received any investigational drug within 4 weeks prior to the first dose of study treatment.
8. The last dose of radiation or anti-tumor therapy (chemotherapy, targeted therapy, immunotherapy, or tumor embolization) was within 3 weeks prior to receiving the first dose of study treatment; the last dose of nitrosourea or mitomycin C treatment was within 6 weeks prior to receiving the first dose of study treatment.
9. Received immunosuppressants within 4 weeks prior to the first dose of study treatment, excluding local glucocorticoids administered by nasal, inhaled, or other topical routes, or systemic glucocorticoids of physiological doses (no more than 10 mg/day of prednisone or equivalents).
10. Received any live attenuated vaccine within 4 weeks prior to the first dose of study treatment, or is scheduled to receive the live attenuated vaccine during the study period.
11. Received major surgery (craniotomy, thoracotomy, or laparotomy) within 4 weeks prior to the first dose of study treatment, or has unhealed wounds, ulcers, or fractures.
12. Active, known, or suspected autoimmune disease (see Appendix 6) or previous medical history of these diseases within 2 years (patients with vitiligo, psoriasis, alopecia, or Graves' disease not requiring systemic treatment, hypothyroidism only requiring thyroid replacement, or type I diabetes only requiring insulin can enroll).
13. Known history of primary immunodeficiency diseases.
14. Known active pulmonary tuberculosis.
15. Known history of allogeneic organ transplantation or allogeneic hematopoietic stem cell transplantation.
16. Known to be allergic to any ingredients of monoclonal antibodies.
17. Uncontrolled concurrent diseases including but not limited to:

- HIV-infected patients (positive anti-HIV antibody).
- Active or poorly controlled severe infections.
- Symptomatic congestive heart failure (NYHA Class III–IV) or symptomatic or poorly controlled arrhythmia.
- Uncontrolled hypertension (systolic blood pressure ≥160 mm Hg or diastolic blood pressure ≥100 mm Hg) despite standard treatment.
- Any arterial thromboembolic events that occurred within 6 months prior to enrollment, including myocardial infarction, unstable angina, cerebrovascular accident, or transient cerebral ischemic attack.
- Life-threatening hemorrhagic events or grade 3–4 gastrointestinal/variceal hemorrhage requiring blood transfusion, endoscopy, or surgical treatment within 3 months prior to enrollment.
- A history of deep venous thrombosis, pulmonary embolism, or other serious thromboembolic events within 3 months prior to enrollment (implantable port or catheter-related thrombosis or superficial venous thrombosis is not considered as serious thromboembolisms).
- Uncontrolled metabolic disorders, nonmalignant organ or systemic diseases, or cancer-related secondary diseases that may lead to higher medical risks and/or survival evaluation uncertainties.
- Hepatic encephalopathy, hepatorenal syndrome, or cirrhosis with Child-Pugh grade B or C.
- Bowel obstruction or a history of the following diseases: inflammatory bowel disease or extensive bowel resection (partial colectomy or extensive small bowel resection accompanied by chronic diarrhea), Crohn's disease, and ulcerative colitis.
- Acute or chronic diseases, psychiatric disorders, or laboratory abnormalities that may lead to the following consequences: increased investigational drug-related risks or interference with interpreting trial results, and considered ineligible for participating in the trial by the investigators.

1. Known acute or chronic active hepatitis B (chronic HBV carriers or inactive HBsAg-positive patients can enroll if the HBV DNA <1 × 10^3^ copies/mL), or acute or chronic active hepatitis C [patients with negative hepatitis C virus (HCV) antibody can enroll; HCV RNA test is required for patients with positive HCV antibody, those testing negative can enroll].
2. A history of gastrointestinal perforation and/or fistula without radical treatment within 6 months prior to the enrollment.
3. Known interstitial lung disease.
4. Clinically uncontrollable third spacing, such as pleural effusion and ascites that cannot be controlled by drainage or other methods prior to enrollment.
5. A history of other primary malignant tumors, excluding:

- A history of radical treatment for malignant tumors with no evidence of tumor recurrence for more than 5 years prior to enrollment and with a very low risk of recurrence;
- Adequately treated nonmelanoma skin cancer or lentigo maligna with no signs of disease recurrence;
- Adequately treated carcinoma in situ with no signs of disease recurrence.

1. Pregnant or breastfeeding female patients.

**

Figure. S1.**

**Figure S1.** Study flowchart.

**Figure. S2.**


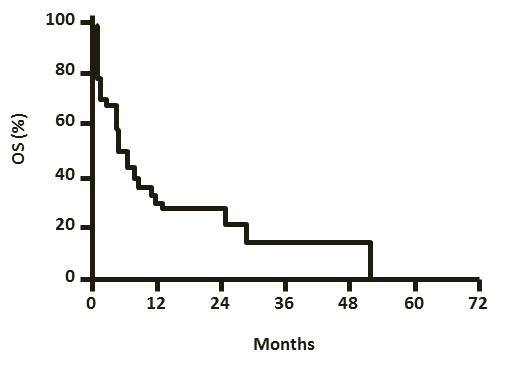


**Figure S2.** Historical control retrospective study of patients with NK/T-cell lymphoma who received standard treatment for relapsed/refractory (r/r) disease. The median overall survival (OS) was 4·8 months.


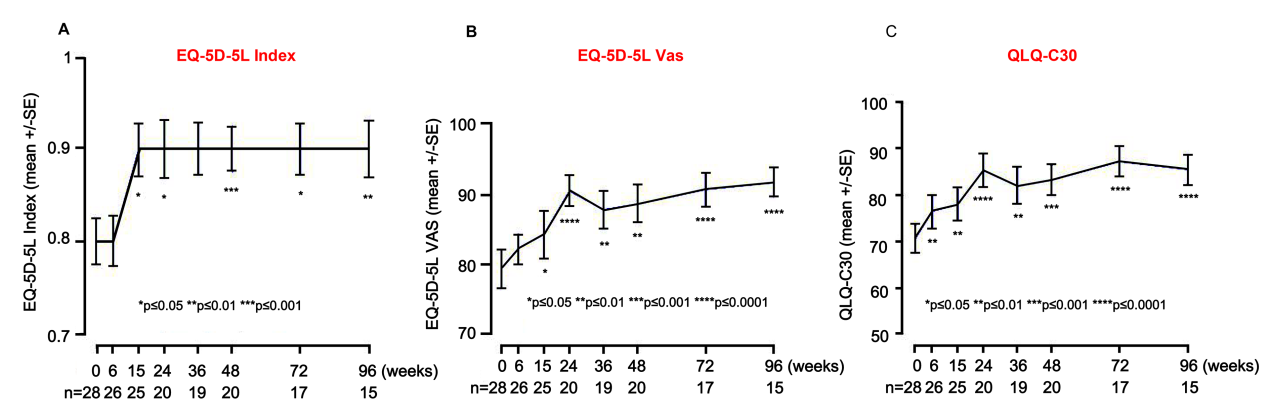
**Figure. S3.**

**Figure S3.** Quality of life evaluated using the EQ-5D-5L and QLQ-C30 questionnaires in patients with NK/T-cell lymphoma treated with sintilimab. ^*^*P* < 0·05, ^**^*P* < 0·01, ^***^*P* < 0·001 vs. baseline.
